# CHOGlycoNET: Comprehensive glycosylation reaction network for CHO cells

**DOI:** 10.1016/j.ymben.2022.12.009

**Published:** 2023-03

**Authors:** Pavlos Kotidis, Roberto Donini, Johnny Arnsdorf, Anders Holmgaard Hansen, Bjørn Gunnar Rude Voldborg, Austin W.T. Chiang, Stuart M. Haslam, Michael Betenbaugh, Ioscani Jimenez del Val, Nathan E. Lewis, Frederick Krambeck, Cleo Kontoravdi

**Affiliations:** a Sargent Centre for Process Systems Engineering, Department of Chemical Engineering, Imperial College London, London, SW7 2AZ, UK; b Department of Life Sciences, Imperial College London, London, SW7 2AZ, UK; c National Biologics Facility, Department of Biotechnology and Biomedicine, Technical University of Denmark, Lyngby, Denmark; d Department of Pediatrics, University of California, San Diego, CA, 92093, USA; e Department of Chemical & Biomolecular Engineering, Johns Hopkins University, Baltimore, MD, USA; f School of Chemical & Bioprocess Engineering, University College, Dublin, D04 V1W8, Ireland; g Department of Bioengineering, University of California, San Diego, CA, 92093, USA; h ReacTech Inc., 810 Cameron Street, Alexandria, VA, 22314, USA

**Keywords:** Protein glycosylation, Chinese hamster ovary cells, Glycoengineering, Systems glycobiology

## Abstract

Chinese hamster ovary (CHO) cells are extensively used for the production of glycoprotein therapeutics proteins, for which N-linked glycans are a critical quality attribute due to their influence on activity and immunogenicity. Manipulation of protein glycosylation is commonly achieved through cell or process engineering, which are often guided by mathematical models. However, each study considers a unique glycosylation reaction network that is tailored around the cell line and product at hand. Herein, we use 200 glycan datasets for both recombinantly produced and native proteins from different CHO cell lines to reconstruct a comprehensive reaction network, CHOGlycoNET, based on the individual minimal reaction networks describing each dataset. CHOGlycoNET is used to investigate the distribution of mannosidase and glycosyltransferase enzymes in the Golgi apparatus and identify key network reactions using machine learning and dimensionality reduction techniques. CHOGlycoNET can be used for accelerating glycomodel development and predicting the effect of glycoengineering strategies. Finally, CHOGlycoNET is wrapped in a SBML file to be used as a standalone model or in combination with CHO cell genome scale models.

## Highlights

• Host glycosylation network determines protein glycoprofile.
• CHO cells used extensively for industrial glycoprotein production.
• We built a comprehensive glycosylation reaction network for CHO cells.
• CHOGlycoNET is informed by 200 glycan datasets.
• It can accelerate model development and design of glycoengineering studies.

## 1 Introduction

Most biotherapeutics are produced in Chinese Hamster Ovary (CHO) cells, which are the workhorse of recombinant protein production in both industrial and academic environments (Walsh, 2018). The design of cell- or process-level glycoengineering strategies to improve the quality profile of glycoprotein-based biotherapeutics is inextricably linked to the underlying glycosylation reaction network (RN) of the host cell line. Depending on the complexity of the protein glycoprofile, a range of significantly diverse RNs has been proposed in different studies, accounting from just 25 up to 40,000 reactions for recombinant immunoglobulin G (IgG) products and host cell proteins (HCPs), respectively (Spahn et al., 2016; Kremkow and LeeGlyco-Mapper, 2018; Krambeck et al., 2017; Jimenez del Val et al., 2011; Hutter et al., 2017). The reconstruction of a RN that is specific to the desired glycoprotein and representative of the machinery of the host cell line is an intricate and time-consuming task. Thus, several algorithms have been developed for the automated RN design or reconstruction, based on experimentally observed glycomic data (Liu and Neelamegham, 2014; Krambeck et al., 2009). Due to competition of glycosyltransferases (GTs) for the same oligosaccharide substrates attached to the glycoprotein, especially among the GTs that reside in the later compartments of the Golgi apparatus, the number of reactions included in the RN disproportionally increases with the complexity of the glycoprofile. However, thousands of possible reactions can be generated, the vast majority of which may not actually occur due to low enzyme levels and steric hindrance imposed by the structural conformation of the glycoproteins.

**Fig. 1:**
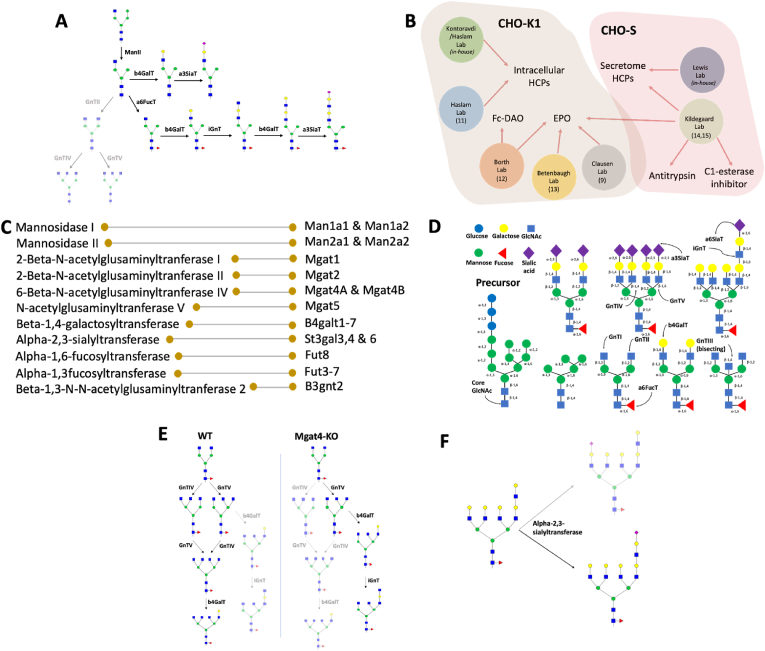
(A) Latent or inactive reactions only observed in knockout cell lines. In the presented pathway, the knockout of GnTII (Mgat2), GnTIV (Mgat4A & Mgat4B) and GnTV (Mgat5) results in the formation of poly-LacNAc mono-antennary glycans. While the reactions for the poly-LacNAc mono-antennary glycan formation can occur in the wild-type cells as well, the flux is directed towards the synthesis of bi-antennary and consequently tri-antennary glycans in the wild-type cells. (B) Datasets utilized for the construction of CHOGlycoNET: Kontoravdi/Haslam Lab (*in-house*), Lewis Lab (*in-house*), Haslam Lab (North et al., 2010), Betenbaugh Lab (Yin et al., 2015), Borth Lab (Bydlinski et al., 2018), Kildegaard Lab (Amann et al., 2018, 2019) and Clausen Lab (Yang et al., 2015). DAO: D-amino acid oxidase, Fc: fragment crystallizable region. (C) Enzymes involved in N-linked glycosylation occurring in the Golgi apparatus and considered for the reconstruction of the dRN. The genes known to express the respective enzymes in CHO cells are also reported. (D) Major glycosyltransferase activity in glycosylation (not CHO specific). (E) Designation of unique reactions in the scenario of Mgat4 knockout that demonstrate the activity of the iGnT enzyme. The comparison between wild-type (WT) and genetically modified cell lines, as illustrated, enables the identification of reactions that are uniquely active in genetically modified cell lines. (F) Preference rules for the processing of iGnT (B3gnt2) products by a3SiaT (St3gal3, St3gal4 & St3gal6) and b4GalT (B4galt1-B4galt7). To effectively reduce the number of reactions considered in CHOGlycoNET, iGnT was assumed to act on a single branch of the substrate and further processing catalysed by a3SiaT and b4GalT was assumed to occur on the branch elongated by the iGnT enzyme.

Large RNs carrying hundreds of muted reactions can decelerate model refinement and gene engineering optimization. Conversely, reduced networks based on wild-type cell lines are likely to omit underlying latent or inactive reactions and may therefore prove inadequate for describing the effect of GT knockout or overexpression studies. For example, the knockout of Mgat2, Mgat4A/B and Mgat5 genes (GnTII, GnTIV and GnTV enzymes, respectively), which are responsible for N-glycan antenna formation via the addition of beta-N-acetylglucosamine (GlcNAc) residues to a tri-mannosyl core, might lead to poly-N-acetyllactosamine (poly-LacNAc) extensions of the monoantennary glycan through the downstream activity of iGnT and b4GalT glycosyltransferases (b3GnT2 and b4GalT1-7 encoding genes, respectively). However, the reaction that would lead to the production of mono-antennary poly-LacNAc extension (Fig. 1A) is not directly observed in wild-type CHO cells that express recombinant erythropoietin (EPO) (Yang et al., 2015) and would therefore be omitted when constructing a RN solely based on wild-type cell data. This problem would equally affect any statistical or stochastic glycosylation models that do not encompass alien glycans missing from the training dataset. Previously, unknown reactions in the IgG glycosylation pathway have been mapped *in silico*, through the utilization of Gaussian graphic models on glycomics data, in an effort to shed light on the hidden reactions of protein glycosylation (Benedetti et al., 2017).

Here, we present CHOGlycoNET, a comprehensive network of glycosylation reactions that accounts for all experimentally observed glycans on recombinant proteins and both intracellular/membrane and secreted HCPs in two major CHO cell lineages, CHO–S and CHO–K1. We collected the largest compendium of CHO cell glycoprofiles, consisting of 200 datasets from seven labs (Fig. 1B) to extract possible latent reactions that could be activated across diverse genetic glycoengineering and metabolic (process) perturbation scenarios for several recombinant glycoproteins and CHO cell HCPs (Fig. 1B). In all studies considered for RN construction, the glycans were identified using mass spectrometry (MS), ensuring the homogeneity of the dataset. The resulting glycosylation RN balances the effects of network size and simultaneously ensures the inclusion of latent reactions and reactions potentially inactive in wild-type cells. Differences between CHO–S and CHO–K1 cells on the reaction network complexity were also identified. In addition, CHOGlycoNET was utilized for the estimation of enzyme distribution solely based on the topology of the reactions involved in the RN and for the identification of critical reactions affecting the extent of glycosylation complexity through dimensionality reduction and machine learning techniques. We envision that CHOGlycoNET will facilitate future designs of glycoengineering strategies for a diverse range of therapeutic proteins and accelerate the design and simulation of glycosylation models.

## 2 Materials & methods

### 2.1 Data curation

Mass spectrometric datasets from 200 glycomics and glycoproteomics samples from CHO cells were considered in this analysis (Fig. 1B). The *Kontoravdi/Haslam Lab* dataset includes the glycoprofile of intracellular HCPs from CHO–K1 IgG-producing cells, under different feeding experiments that incorporate galactose and uridine addition (Supplementary Material 5). The *Lewis Lab* dataset describes the glycoprofile of the secretome from non-producing CHO–S cells, including distinct clones with knockouts on Mgat4 (GnTIV), Mgat2 (GnTII), B3gnt2 (iGnT), B4galt1-3 (b4GalT) and St3gal3,4,6 (a3SiaT) genes (Supplementary Material 5). The *Haslam Lab* dataset includes intracellular HCP data from wild type and mutated CHO Lec cells (North et al., 2010). The *Borth Lab* dataset characterises the effect of b4GalT isoform knockouts on the glycoprofile of recombinantly produced fusion glycoproteins (EPO and D-amino acid oxidase; DAO) (Bydlinski et al., 2018). The dataset from the *Betenbaugh Lab* investigates the effect of Mgat4/Mgat5 overexpression, alongside the expression of the human ST6GAL1 gene expressing the alpha-2,6-sialyltransferase enzyme (Yin et al., 2015). The *Clausen Lab* dataset includes the effect of the knockout of numerous glycosyltransferases on EPO and IgG glycosylation (Yang et al., 2015). Finally, the *Kildegaard Lab* dataset describes the effect of glycoengineering on multiple recombinant glycoproteins and the secretome (Amann et al., 2018, 2019).

In the case of non-exhaustively annotated mass chromatograms, the m/z peaks in question were identified through the GlycoWorkbench software (Ceroni et al., 2008). In total, 265 unique glycan structures, including potential isomers, were identified. Hybrid glycans were not considered in this study. Whilst no minimum distribution threshold was set for qualifying the inclusion of a reported glycan in CHOGlycoNET, oligosaccharides carrying more than eight GlcNAc molecules were excluded from the analysis as they were identified only in trace amounts. Whilst glycoproteomic analysis has shown that glycan structures with >8 GlcNAc molecules can be traced in cellular proteins of CHO cells (Yang et al., 2015), they are not commonly encountered in recombinant therapeutic glycoproteins. The mannosidases and GTs found to be active in CHO cells in CHOGlycoNET are shown in Fig. 1C and D.

### 2.2 Isomer inclusion

The oligosaccharides considered account for all isomers of each experimentally observed bi-, tri- and tetra-antennary glycan that have not been further elongated by iGnT, as the steric hindrance imposed on the GTs is strongly dependent on the isomer structure and can determine the kinetics of the reaction and ultimately the final isomer distribution. To identify reactions that are only activated in glycoengineered cell lines, we assumed that no bi-, tri-, and tetra-antennary glycans of the wild-type cell lines were products of the iGnT enzyme. To elaborate, it was assumed that iGnT could act on a galactosylated substrate only when GnTI, GnTII, GnTIV and GnTV could not further process the glycan or when the enzymes were genetically silenced. This enabled the comparative analysis between the wild type and the glycoengineered cell lines and the designation of the latent reactions (Fig. 1E). It is important to note that previous research on the analysis of the N-glycan pool carrying 6-LacNAc molecules in wild type CHO (Pro ‾5) cells grown in suspension and using MALDI-TOF/TOF MS/MS has identified a bi-antennary oligosaccharide with five LacNAc molecules on one arm and one on the other arm as a major isomeric structure (North et al., 2010). Whilst the iGnT elongation of bi- and tri-antennary glycans can occur in wild type CHO cells, the actual abundance of such structures requires additional experiments to more accurately quantify the detailed distribution, which could be heavily dependent on the examined protein and cell line; thus, it was excluded from the reaction network analysis of the wild type cells. Moreover, this assumption is not expected to have a major effect on the identification of the global network, as the iGnT elongation reactions not considered in the wild type cells were inevitably included in the analysis of the knockout cell lines. Finally, b4GalT and a3SiaT show no known preference on the extension of tri- and tetra-antennary glycans that carry LacNAc molecules, and therefore only one isomer was included for these reactions. The galactosylation and sialylation of the poly-LacNAc branch was prioritized in the isomer selection over the non-LacNAc-elongated branches (Fig. 1F).

### 2.3 Computational tools

#### 2.3.1 Network construction

Briefly, GLYMMER (ReacTech) enables the estimation of glycosylation enzyme concentrations based on the experimentally observed glycoprofile, also supporting the direct fitting of mass spectra (Krambeck et al., 2009). The generation of the reaction network based on pre-defined rules of enzymatic promiscuity is also part of GLYMMER. To that end, the GLYMMER functionality was utilized to identify minimum reaction networks based on the experimentally observed glycoprofile, through the newly introduced *Lumping* function. The *Lumping* function identifies reactions essential for synthesizing the experimentally observed glycan structures, choosing the dominant reaction pathways based on their respective reaction rates. This process adds to the experimentally observed essential oligosaccharides whichever intermediate oligosaccharides are required to produce a connected network of reactions, thus forming minimum-RNs (mRNs). However, the resulting network is somewhat deficient in that there are more reactions known to be taking place between certain pairs of oligosaccharides in the minimum network than just the minimum reactions needed to connect the network. These additional reactions are added to the deficient network to result in a complete network. In practice, this addition does not add many more reactions to the network but more accurately represents the dependencies of model solutions on individual enzyme activities. Cytoscape v3.8 was used for network visualization and hierarchical representation (Shannon et al., 2003). Finally, the network was exported to an SBML file using the COBRApy package in Python 3.7.3 (Supplementary Material 7).

#### 2.3.2 Dimensionality reduction and machine learning

All analyses were performed in Python 3.7.3 using various packages, most notably pandas, umap-learn and sklearn. Dimensionality reduction was performed with both Principal Component Analysis (PCA) and Uniform Manifold Approximation and Projection (UMAP). The UMAP configuration included a number-of-neighbours equal to 15, minimum distance set to zero and the use of the Euclidean metric. Following UMAP reduction, k-means was used for clustering the data in the reduced dimensionality. For the interpretation of UMAP embeddings and feature importance towards dimensionality reduction (McInnes and Healy, 2018), four models, namely least absolute shrinkage and selection operator (LASSO), Ridge, Elastic Nets and Random Forests were used, as these models are well-established for feature selection (Cai et al., 2018). The relevant code can be found in Supplementary Material 6.

All Supplementary Material can be found in CHOGlycoNET, Mendeley Data, DOI:10.17632/pph9ksfvjd.1

## 3 Results

### 3.1 RN reconstruction and curation

**Fig. 2:**
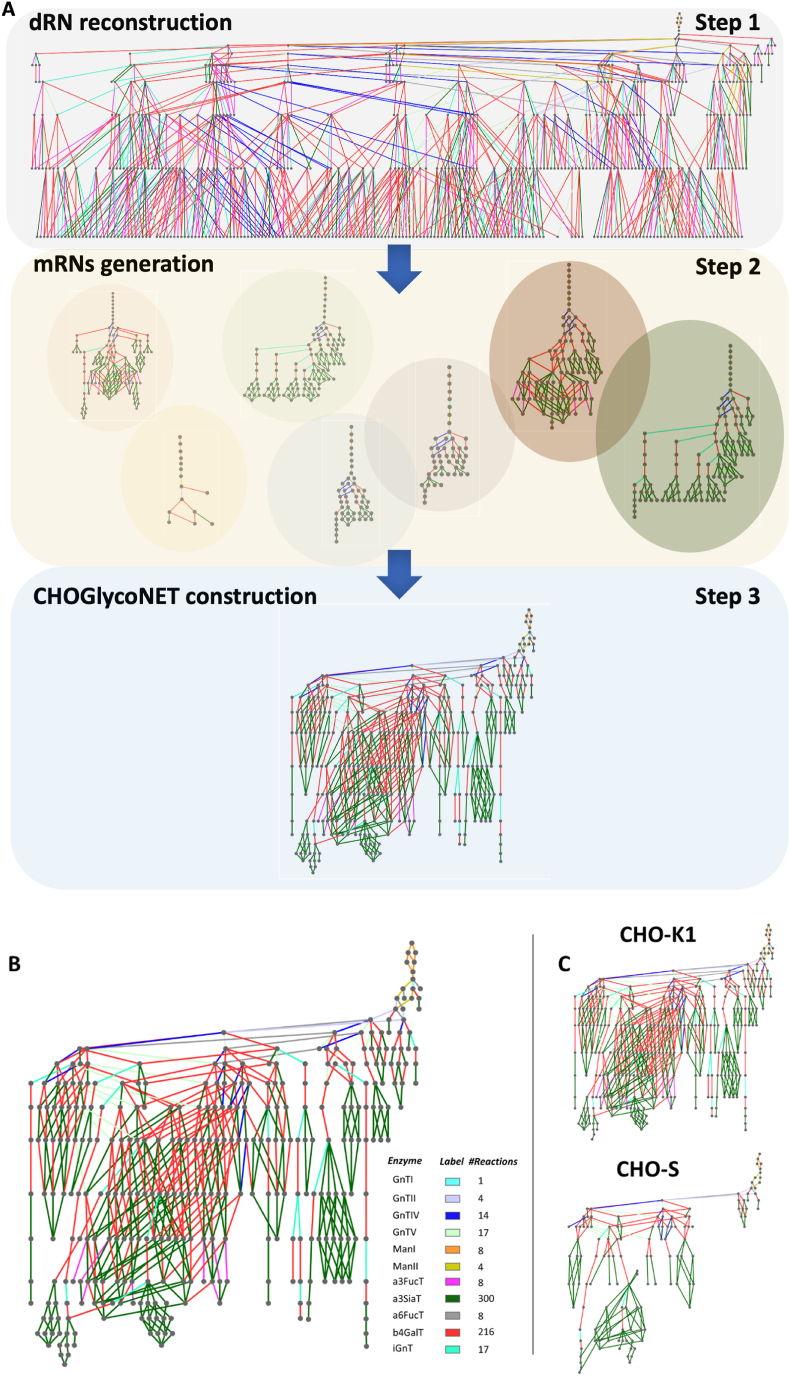
(A) Steps for the construction of the CHOGlycoNET. Only 1000 reactions of the dRN are shown for illustration purposes. *Step 1*: The process starts with the reconstruction of the dRN, a detailed glycosylation reaction network that describes the activity of 11 enzymes and 22 genes for the generation of ∼83,000 plausible reactions and ∼29,500 oligosaccharides. The dRN is used as a standard template for the generation of the individual mRNs for every examined dataset. *Step 2*: A unique mRN is generated for all the experimentally measured sets of glycans using the *Lumping* algorithm. The mRNs describe the minimum set of reactions essential for the synthesis of the experimentally observed glycans. *Step 3*: The individual mRNs are finally concatenated to produce the CHOGlycoNET. The concatenation step includes the one-by-one comparison of the mRN networks and ensures that the formed CHOGlycoNET is a superset of all the mRNs. (B) The resulting CHOGlycoNET for both CHO–K1 and CHO–S cells as identified from all the datasets included in this study. The network consists of 597 reactions and 326 oligosaccharides that are generated from the activity of 11 enzymes. The different colours of the reactions indicate the activity of a different enzyme, as shown in the respective legend. (C) RNs with the minimum number of reactions to describe the CHO–K1 and the CHO–S networks separately. Missing edges between nodes in host-cell specific networks indicate the absence of corresponding reactions compared to the full-scale CHOGlycoNET.
• **Step 2:** Using the *Lumping* algorithm developed in GLYMMER (Krambeck et al., 2009), a minimum reaction network (mRN) was constructed for each experiment considered in this analysis (Fig. 2A, Step 2), using the dRN as a template. The mRN for each experiment is a sub-network of the generic dRN and describes the minimum number of reactions necessary for the synthesis of the experimentally observed set of glycans. The length of each mRN is dependent on the complexity of the oligosaccharides included in each experimental dataset. The set of enzymes considered, alongside their specificity rules, were common across the mRNs. The enzymate specificity rules were adapted from Krambeck et al. (2009) and can be found in the Supplementary Material. A total of 200 mRNs were generated and were further combined for the reconstruction of the CHOGlycoNET as described in Step 3.
• **Step 3:** Lastly, all mRNs constructed with the *Lumping* algorithm were concatenated to form CHOGlycoNET. The reactions that were unique in each examined network were included in CHOGlycoNET (Fig. 2A, Step 3). As an example, the mRN of an EPO-producing CHO–K1 cell line, a product of *Lumping* of the dRN based on the experimentally observed glycoprofile, was reduced to 88 oligosaccharides and 141 reactions. It is important to note that GnTIII (Mgat3 gene), which is responsible for the addition of bisecting N-acetylglucosamine and is inactive in unmodified parental CHO cells (Yang et al., 2015), was excluded from this analysis.

The construction of CHOGlycoNET includes three major steps as detailed below:

• **Step 1:** Initially, a detailed reaction network (dRN) with 29,443 oligosaccharides and 82,929 reactions was generated, accounting for all possible reactions and glycans and based on the 11 considered glycosyltransferases/mannosidases (corresponding to 22 genes) shown in Fig. 1C and their specificity rules. A generic structure of the dRN is shown in Fig. 2A, Step 1.

CHOGlycoNET is exported in an SMBL file (Supplementary Material 7: “choglyconet.xml”) and can therefore be further easily utilized by other researchers as a standalone model or in combination with CHO genome scale models through, i.e., COBRApy or the COBRA Toolbox.

### 3.2 CHOGlycoNET describes the diverse glycan biosynthetic steps in CHO cells

The resulting CHOGlycoNET includes 597 reactions and 326 oligosaccharides (see Supplementary Material 1 for full set of reactions). The majority of reactions are catalysed by b4GalT and a3SiaT (Fig. 2B). The small number of reactions assigned to earlier processing enzymes indicates the consistency of reactions in the first steps of glycosylation for different proteins and across CHO–S and CHO–K1 host cells.

CHOGlycoNET includes all reactions necessary for the generation of the 326 oligosaccharide structures identified among the 200 considered samples. Importantly, it only adds 61 intermediate structures to the experimentally identified structures to enable the generation of the observed glycoprofiles. The small number of additional glycans that are necessary for CHOGlycoNET construction is mostly attributed to the almost exhaustive examination of the various knockout scenarios that, as previously mentioned, give prominence to latent reactions. The network can serve for evaluating both the qualitative and quantitative effects of gene engineering on glycoprofile microheterogeneity. Moreover, CHOGlycoNET can inform mathematical model development for describing protein glycosylation *in silico*. The inclusion of data from experimental glycoengineering studies as well as wild-type CHO cell HCP glycans ensures that CHOGlycoNET can account for alien glycans that are absent from the unmodified parental cell line data but occur after glycoengineering. This means that models formulated based on CHOGlycoNET and adequately trained using relevant experimental data, such as transcriptomic and/or proteomic analysis of wild-type and glycoengineered cells, could, in theory, account for off-target glycoengineering effects.

### 3.3 CHOGlycoNET highlights cell line-specific protein glycosylation reactions

CHO–K1 cells generally presented RNs with higher complexity than CHO–S cells, with 594 reactions of CHOGlycoNET active in the former compared to only 192 active reactions in the latter (Fig. 2C). Differences in the maturation levels of IgG glycans between the two cell lines have been previously reported (Reinhart et al.Bioprocessing of Recombinant CHO-K1CHO-DG44CHO, 2019). *In-house* glycomic data from the intracellular HCPs of both cell lines indicate a higher degree of microheterogeneity and further processed glycans in the CHO–K1 cells (*data not shown*). Apart from the plausible differences in the complexity of the RNs due to the glycosylation machinery of the two cell lines, e.g., enzyme levels, these differences could be originating from the diversity of the datasets. While the CHO–S dataset examines a range of recombinant proteins and HCPs (Fig. 1B), the CHO–K1 data includes a thorough screening of GT knockouts (*Clausen Lab* – CL – dataset), presented in Yang et al. (2015). This inclusion contributes to the designation of latent reactions and alternative reaction pathways and therefore the enrichment of the CHO–K1 RN. GT knockout experiments are included in the CHO–S dataset as well, albeit at a smaller scale. Additionally, the CHO–S data, while highly diverse, accounts for ∼13% of the total samples. This is another factor that could contribute to the resulting simplicity of the CHO–S reaction network. Regardless of the differences between the individual networks, CHOGlycoNET is proposed for both parental cell lines and covers a plausible higher complexity of the CHO–K1 network.

**Fig. 3:**
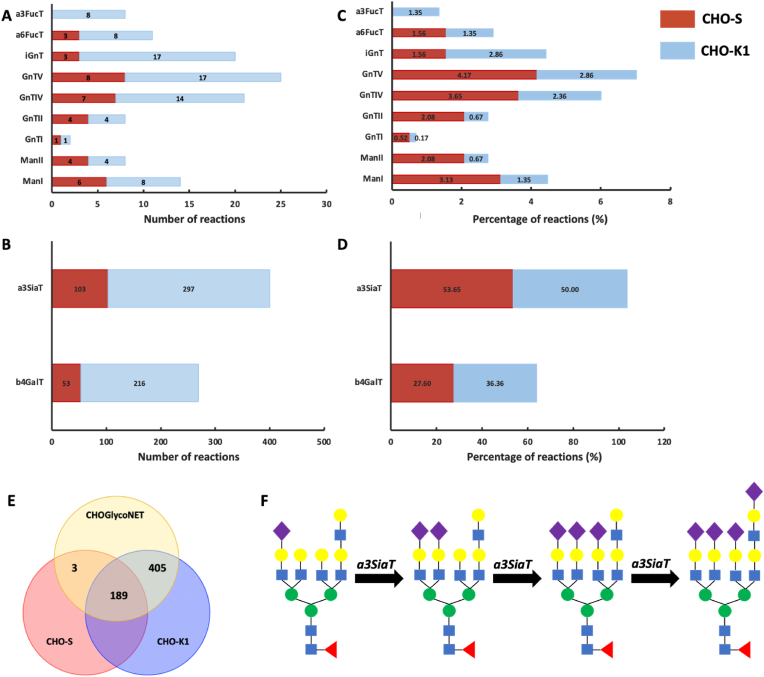
Evaluation of the contribution of each enzyme in the network of CHO–S and CHO–K1 cells through the absolute number of reactions catalysed by each enzyme (A & B) and the percentage of reactions against the total number present in each cell-line specific network (C & D). a3SiaT and b4GalT are examined separately due to the considerably higher number of reactions compared to the rest of the enzymes. (E): Overlaps between the CHOGlycoNET, CHO–S and CHO–K1 networks. (F): The only 3 reactions missing from the CHO–K1 RN and present in the CHO–S.

Interestingly, while the number of reactions catalysed by each enzyme exhibits considerable differences between the two cell-lines (Fig. 3A and B), the relative percentage is more equally distributed (Fig. 3C and D). This observation is more prominent in the case of a3SiaT and b4GalT, where the number of reactions for CHO–S cells is 103 and 53 for a3SiaT and b4GalT respectively, and 297 and 216 for the CHO–K1 cell-line. However, the a3SiaT-catalysed reactions are ∼50% of the total RN for both cell-lines, while the respective percentages for b4GalT are 28% and 36% for CHO–S and CHO–K1, respectively. The high combined number of reactions catalysed by a3SiaT and b4GalT showcase the dependence of glycan complexity on the two enzymes. The b4GalT4 isomer has been found to regulate glycans’ branching in the N-linked glycosylation of a recombinantly produced human chorionic gonadotropin (hCG) protein (McDonald et al., 2014a). It is also important to note that most reactions present in the CHO–K1 and not in the CHO–S cell-line are catalysed by the a3SiaT and b4GalT enzymes. The overlap between the three datasets is shown in Fig. 3E. The three sialylation reactions that are only active in the CHO–S RN are shown in Fig. 3F.

### 3.4 Data type effects on the CHOGlycoNET

CHO HCP glycomic datasets were obtained using various mass spectrometry-based techniques, therefore potential differences in the datasets could be attributed to variations in the experimental methodology. A liquid chromatography (LC) step prior to MS analysis can enable the differential elution of individual glycans, thus improving the identification of isomeric structures (Veillon et al., 2017). Alternatively, isomeric structures can also be differentiated in MALDI-based analysis by MS/MS fragmentation (North et al., 2010). LC-MS was used to analyse the *Lewis & Kildegaard Lab* data (abbreviated as LL and KL, respectively), electrospray ionization-MS (ESI-MS) for the *Borth Lab* (BL) data and the remaining datasets were obtained with matrix assisted laser desorption ionization-time of flight-MS (MALDI-TOF-MS). In addition, some analyses include sample derivatisation methods which can increase glycan integrity during ionization (e.g., permethylation) and therefore improve sensitivity. Therefore, owing to the discrepancies in the sensitivity of the different instruments used for the generation of the data included in this study, we did not set a minimum detection limit for a glycan to be considered in CHOGlycoNET. Thus, all glycan structures reported in the original studies were included in the analysis.

**Fig. 4:**
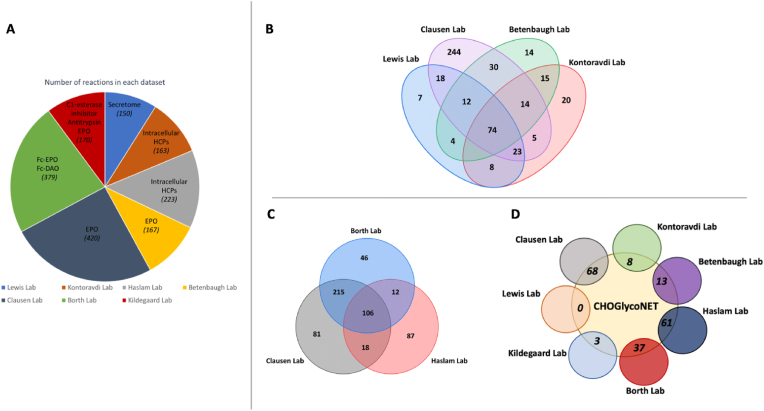
(A) Number of the total reactions present sub-RNs built for each individual dataset. (B) The overlap of the contribution of each sub-RN towards the reconstruction of the CHOGlycoNET. (C) Overlap of the contribution between the three datasets that result in the largest sub-RNs. (D): Unique reactions contributed by each dataset towards the CHOGlycoNET construction.

Each dataset contributes a different number of reactions towards CHOGlycoNET construction (Fig. 4A). The sub-RNs, i.e., the reaction networks specific to each individual dataset, were also reconstructed in order to evaluate the contribution of each dataset. Overlaps between the reactions present in each dataset were identified (Fig. 4B). Reasonably, the elaborate analysis (MALDI-TOF-MS) of the effect of GT knockouts on EPO glycosylation from the CL dataset, and the site-specific glycomics (LC-ESI-QTOF-MS/MS) of the EPO & DAO Fc fusion glycoproteins from the BL dataset, result in the largest sub-RNs. Interestingly, the aforementioned two sub-RNs share 321 reactions (∼85% of the total number of reactions of the BL dataset), while a total of 333 reactions are common amongst the three largest datasets (Fig. 4C). However, the number of unique reactions that each of the three largest sub-RNs contribute towards the CHOGlycoNET reconstruction is considerably lower (Fig. 4D). In fact, the CL-specific sub-RN contributed 68 reactions, while the BL-specific sub-RN contributed another 37. Importantly, a subset of 407 reactions (∼70%) of the CHOGlycoNET is common across more than one dataset. CHOGlycoNET therefore exhibits minor dependencies on the individual datasets. Moreover, the LL dataset was found to not contribute any unique reactions towards the global network, despite the incorporation of several gene knockout experiments that are important for identifying latent reactions. Both the LL and KL analyses that were conducted on CHO–S cells contributed the least number of unique reactions to CHOGlycoNET, further supporting the observation that CHO–S cell lines exhibit less complex and divergent reaction network. The inclusion of the LL dataset in the analysis is important for demonstrating the completeness of CHOGlycoNET and its applicability towards describing complex glycosylation profiles such as the one extracted from the secretome.

### 3.5 Mannosidase and glycosyltransferase distribution based on network structure

The network representation depicted in Fig. 2B is based on increasing glycan complexity. Twenty-three levels of complexity can be identified based on the CHOGlycoNET structure (Fig. 2B). As glycosylation is a sequential process, increasing glycan complexity is related to the distance that the protein molecule has covered within the Golgi apparatus. Thus, the number of reactions catalysed by each enzyme and for each individual level can be used to investigate the likely sequence of mannosidases and glycosyltransferases and their distribution along the Golgi length. The distribution in each level was calculated as the ratio of reactions occurring in the specific level over the total number of reactions catalysed by each enzyme, while the Golgi length (distance from level 1 to level 23) was normalized between 0 and 1. It is important to note that the enzyme sequence presented herein does not account for activity, assumes that glycoproteins spend an equal amount of time in each Golgi compartment and is therefore not meant to indicate enzyme localisation.

**Fig. 5:**
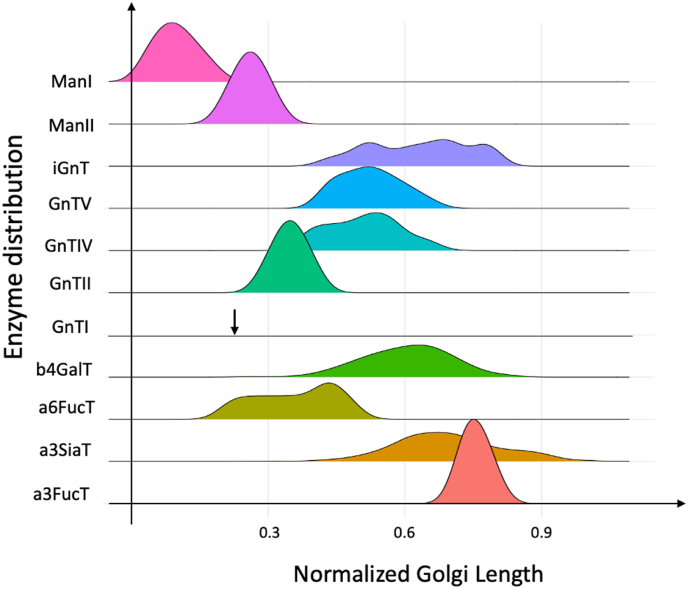
Distribution of each enzyme along the length of the Golgi apparatus (x-axis shows normalized organelle length). Note that GnTI has no distribution because the enzyme catalyses only a single reaction in the identified network. An arrow is used to indicate GnTI localisation within the normalized Golgi apparatus.

As shown in Fig. 5, enzymes catalysing early steps of the glycosylation reaction network (ManI, ManII and GnTII) demonstrate normal distributions (a distribution of GnTI does not exist as the enzyme catalyses a single reaction in CHOGlycoNET). The aforementioned enzymes are less promiscuous and relate to a part of the RN that is conserved and convergent. Therefore, as expected, the number of reactions that the enzymes participate in is relatively small, ranging between four and eight. Similarly, a3FucT presents a normal distribution in the latter part of the Golgi, following a reasonable pattern, as the main function of the a3FucT includes the addition of fucose molecules on highly branched glycans that are encountered along several locations of the Golgi apparatus. Interestingly, GnTIV and GnTV present overlapping distributions with high levels of similarity, potentially owing to their similar function in glycan branching. In addition, b4GalT and a3SiaT show high degrees of overlap and a wide normal distribution, being present in >50% of the Golgi apparatus length. iGnT is co-localized with b4GalT and a3SiaT and presents a multimodal distribution, unlike most glycosyltransferases, probably due to its ability to act on any substrate that is terminally galactosylated. Lastly, a6FucT presents a mostly bimodal distribution between 0.2 and 0.4 of the normalized Golgi length, followed by the highest peak at approximately 0.45 and the complete depletion of enzyme levels thereafter. Overall, the results broadly indicate a normal distribution of the enzymes, except for a6FucT and iGnT enzymes, partially supporting the mathematical formulations using the Golgi maturation models (Hossler et al., 2007). The proposed sequence is in agreement with experimental findings of glycosidase and glycosyltransferase localisation experiments in CHO (Velasco et al., 1993) and HeLa cells (Rabouille et al., 1995; Hassinen et al., 2010). They are also in line with computational predictions of enzyme activity along the Golgi length generated using a kinetic glycosylation model for CHO cells (Arigoni-Affolter et al., 2019). A recent experimental investigation of glycosyltransferases localisation in the Golgi organelle of human cells revealed that GnTII is localized downstream of GnTI and b4GalT7, while GnTIV was found to reside in earlier parts of the Golgi apparatus compared to the aforementioned enzymes (Tie et al., 2018). However, as also mentioned in the original study, these observations relate to cells in which the respective genes had been overexpressed, which may have perturbed the natural localisation of the enzymes (Cosson et al., 2005).

### 3.6 Addition of β4-GlcNAc and subsequent galactosylation regulate the extension of the observed glycosylation network

In order to evaluate the importance of each of the 597 identified reactions in the shaping of the observed glycosylation network for each of the considered samples, a dimensionality reduction technique, namely UMAP, was employed for capturing data variance on CHOGlycoNET. UMAP is a non-linear dimensionality reduction method, competitive to the well-established t-SNE algorithm (van der Maaten and Hinton, 2008), that ensures the preservation of the data global structure in the reduced dimensionality (McInnes and Healy, 2018) and with major applications in single cell data visualization (Becht et al., 2019).

**Fig. 6:**
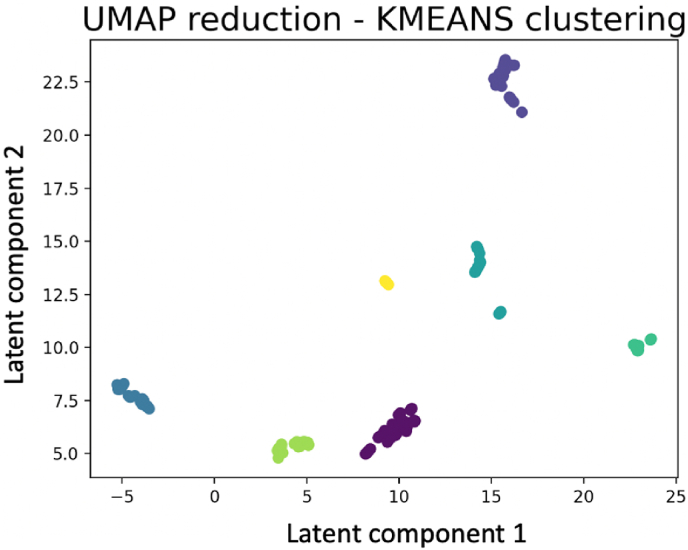
Reduced dimensionality of CHOGlycoNET using UMAP. Following dimensionality reduction, k-means clustering (n = 7) was employed to group the observed clusters of samples. Colouring of samples indicates different clusters.

A matrix of *AxB* size, with *A* being the number of observations (samples) and *B* the number of reactions in CHOGlycoNET was used for dimensionality reduction. Each row of the matrix represents a sample and each column a reaction of CHOGlycoNET. Therefore, the matrix dimensions were *200x597*. The matrix describes whether a reaction is active in each sample; if a reaction is active then the value of the point for the sample (row) and this reaction (column) would be 1 and if the reaction is inactive the respective value would be 0. Next, the UMAP algorithm was used to reduce the dimensionality of the dataset and identify latent components that efficiently describe the variance of the system. The matrix can be found in the supplementary material (SupMat2_Partition.csv). As shown in Fig. 6, CHOGlycoNET was successfully reduced to two latent components that offered a distinct clustering of the considered samples. The clustering of samples was not found to be dependent on 1) cell lineage, 2) lab of origin or protein analysed, as shown in Fig. S1.

However, unlike dimensionality reduction techniques such as principal component analysis (PCA), UMAP does not provide loadings of the features from the original dimensionality, therefore not revealing the contribution of each initial feature towards the calculation of the latent components. Notably, the use of PCA for dimensionality reduction resulted in a relatively low capture of variance, with only 65% explained by 5 PCA components. To overcome the limitation imposed by the lack of UMAP-based feature importance, four machine learning models, namely Lasso, Ridge, ElasticNet and Random Forests were employed to capture the transition from the original to the reduced dimensionality. The aforementioned algorithms are powerful tools for feature selection (Cai et al., 2018), meaning the identification of the most important features for calculating the value of the targeted label. Notably, apart from Random Forests, the remaining estimators are linear models that were expected to perform reasonably well due to the linear Euclidean metric used for UMAP reduction. More specifically, the models were trained on the original matrix *AxB* (inputs) and with the target variables being the latent components as calculated from the UMAP reduction (outputs). Consequently, the models were trained in order to mimic the UMAP reduction of dimensionality between the original dataset and the latent components and were subsequently used to extract important features. As shown in Table 1, all models achieved high R^2^ and low mean squared errors (MSE), with Random Forests outperforming the rest of the estimators during the cross-validation and hyper-parameter tuning. The R^2^ on the test set was calculated at ∼0.96, indicating good generalization capabilities from the tuned Random Forest. Whilst nested cross-validation is usually employed for model selection, it can be unnecessarily expensive for most applications and was therefore avoided in the current study (Wainer and Cawley, 2021). A repeated k-fold cross-validation with 10 splits and 5 repeats was used for model selection on 70% of the dataset, whilst 30% of the dataset was used for testing. Finally, following the evaluation of models through CV, the best model was trained on the entire dataset. The models were used to predict both UMAP embeddings simultaneously.

**Table 1:** Results of machine learning models used for capturing UMAP reduction.

Model	CV score (R^2^/MSE)
Lasso	0.818/7.037
Ridge	0.847/6.125
ElasticNet	0.805/7.731
Random Forest	0.902/3.759

Following its identification of the best performing model, a Random Forest was subsequently tuned on the entirety of the dataset. Tuning was performed through a repeated k-fold cross validation (10 splits, 5 repeats) as well. As the Random Forest model was used for estimating the latent UMAP components utilizing the original dataset, the resulting feature importance values of the Random Forest were used to represent the contribution of each reaction to the formation of the UMAP components. Reactions R38 and R53 were found to contribute the most towards the variance between different samples, demonstrating a key node (reaction) of the RN identified for each sample. Based on feature importance derived from the tuned Random Forest model, GnTIV activity on A2G0F and the subsequent galactosylation of the product through b4GalT showed the highest importance among the reactions in CHOGlycoNET (Table 2). Interestingly, a6-fucosylation of the early A1G0 glycan was also designated as an important reaction characterising the complexity of the glycosylation network. R16 was almost perfectly correlated with R23 (Pearson correlation coefficient ∼0.97) that includes the addition of GlcNAc to A1G0F (R16 product) for the formation of G0F through GnTII activity. However, R16 shows no correlation with R38 and R53 (Pearson correlation ∼ −0.1). Therefore, the pathway of sequential reactions connecting A1G0 to A3G1F (product of R53) was found to considerably contribute to network complexity.

**Table 2:** Top reactions as identified by the Random Forest estimator.

Reaction	Substrate	Product	Enzyme	Importance
R38			GnTIV	0.336
R53			b4GalT	0.209
R16			a6FucT	0.103

## 4 Discussion

N-linked glycosylation is a critical post translational modification of recombinant glycoproteins, significantly affecting molecule activity, structure and immunogenicity. Glycosylation is following a vast and complex reaction network, with thousands plausible reaction pathways leading to the same terminal glycan product. Elucidating the details of the glycosylation RN in CHO, the most widely used mammalian platform for recombinant proteins production in both industry and academia, would enable the design of calibrated glycoengineering strategies for further improving the quality of therapeutic proteins.

First, the general reaction network with 29,443 plausible oligosaccharides and 82,929 reactions was reduced to a network of 326 structures and 597 reactions, achieving a reduction of 90x and ∼140x for the structures and reactions, respectively. A total of 200 different glycan datasets from 7 different labs and different glycoproteins, including both intracellular and secreted HCPs, were utilized in order to construct the CHOGlycoNET. The inclusion of several comprehensive glycoengineering experiments, enables the identification of several plausible glycan structures that would not have been otherwise detected. Additionally, CHOGlycoNET, carrying compressed information from 200 datasets, can considerably accelerate the development and simulation of mechanistic and stochastic glycosylation models, by alleviating the need for glycosylation network construction and reduction of necessary times for model optimization due to the reduced size. In addition, CHOGlycoNET is built on data from both CHO–K1 and CHO–S cell lines, extending the applicability of the network to both hosts.

The hierarchical reconstruction of the glycosylation network based on the minimum number of reactions and intermediate structures necessary to produce the experimentally observed glycans, enabled the identification of 23 reaction levels for the synthesis of the most complex glycan observed in the CHOGlycoNET. Furthermore, glycoenzymes distribution was estimated based on the different reaction levels of the network. Enzyme distribution was found to follow expected patterns for all glycosyltransferases according to literature, improving confidence in network structure. Whilst enzyme localisation can be cell line specific (Colley, 1997), even between different CHO cell lines, the presented distribution was constructed based on data from several different clonal CHO cell lines. The co-distribution of b4GalT and a3SiaT in the medial and late parts of the Golgi cisternae has been reported before for several cell lines (Rabouille et al., 1995; Schaub et al., 2006), in addition to observed sialyltransferase/galactosyltransferase heteromers identified in mammalian cells (Khoder-Agha et al., 2019). ManII and GnTI were found localized in the early parts of the cisternae, following a similar profile with previous reports of the enzymes being present mostly on the medial compartment (Rabouille et al., 1995). The development of CHOGlycoNET consolidates the information content of 200 different samples, providing a comprehensive reaction network that can be used to develop mechanistic, kinetic (e.g. (Jimenez del Val et al., 2011),) or stoichiometric (e.g. (Hutter et al., 2017),), glycosylation models. The proposed enzyme distribution can further aid model parameterisation, which is a challenging and computationally expensive task.

Following the reconstruction of CHOGlycoNET, the utilization of machine learning-mediated dimensionality reduction (Random Forest based interpretation of UMAP latent components) enabled the identification of key reactions in the network. Interestingly, the most notable reactions regulating RN complexity for each of the samples considered were found to be R38, R53 and R16. b4GalT4, one of the b4GalT isoforms catalysing reaction R53, has been previously characterised as a major enzyme for the regulation of glycans branching in CHO cells (McDonald et al., 2014b). We envision that the identified reactions and enzymes can be used to manipulate the complexity of the glycosylation network towards the production of more uniform glycoprofiles in recombinantly produced proteins.

## 5 Conclusion

Herein, we presented a glycosylation reaction network, namely Comprehensive Glycosylation Reaction Network for CHO cells, or CHOGlycoNET, that incorporates all the possible reactions occurring in CHO–K1 and CHO–S cells, as derived from a dataset of 200 glycomic and glycoproteomic profiles from different CHO cell lines obtained by seven research groups. This extensive glycoprofile dataset further covers various recombinant and host cell proteins. Our results demonstrated that CHOGlycoNET can be used to describe the glycosylation of the majority of glycoproteins in both CHO–S and CHO–K1 cells. The increased complexity of glycoproteins considered in the datasets and diversity of glycoengineering and cell culture conditions applied in the experimental datasets has enabled the identification of the minimum set of reactions that are necessary for describing the CHO cell glycosylation system. We envisage that CHOGlycoNET will find applications in the efficient design of glycoengineering strategies as well as the accelerated development of predictive *in silico* glycosylation models.

## Author statement

Pavlos Kotidis: conceptualisation, methodology, formal analysis, Visualization, Writing - Original Draft.

Roberto Donini: investigation, Writing - Review & Editing.

Johnny Arnsdorf: investigation.

Anders Holmgaard Hansen: investigation.

Bjørn Gunnar Rude Voldborg: investigation, resources.

Austin W.T. Chiang: methodology, Writing - Review & Editing.

Stuart M. Haslam: investigation, resources, supervision, Writing - Review & Editing.

Michael Betenbaugh: resources, Writing - Review & Editing.

Ioscani Jimenez del Val: methodology, Writing - Review & Editing.

Nathan E. Lewis: methodology, Writing - Review & Editing.

Frederick Krambeck: methodology, software, resources, Writing - Review & Editing.

Cleo Kontoravdi: conceptualisation, methodology, supervision, Writing - Review & Editing.

## Data Availability

All data have been made available on Mendeley Data (DOI:10.17632/pph9ksfvjd.1).

## References

[bib1] Amann T. (2018). CRISPR/Cas9-Multiplexed editing of Chinese hamster ovary B4Gal-T1, 2, 3, and 4 tailors N-glycan profiles of therapeutics and secreted host cell proteins. Biotechnol. J..

[bib2] Amann T. (2019). Glyco-engineered CHO cell lines producing alpha-1-antitrypsin and C1 esterase inhibitor with fully humanized N-glycosylation profiles. Metab. Eng..

[bib3] Arigoni-Affolter I. (2019). Mechanistic reconstruction of glycoprotein secretion through monitoring of intracellular N-glycan processing. Sci. Adv..

[bib4] Becht E. (2019). Dimensionality reduction for visualizing single-cell data using UMAP. Nat. Biotechnol..

[bib5] Benedetti E. (2017). Network inference from glycoproteomics data reveals new reactions in the IgG glycosylation pathway. Nat. Commun..

[bib6] Bydlinski N. (2018). The contributions of individual galactosyltransferases to protein specific N-glycan processing in Chinese Hamster Ovary cells. J. Biotechnol..

[bib7] Cai J. (2018). Feature selection in machine learning: a new perspective. Neurocomputing.

[bib8] Ceroni A. (2008). GlycoWorkbench: a tool for the computer-assisted annotation of mass spectra of glycans. J. Proteome Res..

[bib9] Colley K.J. (1997). Golgi localization of glycosyltransferases: more questions than answers. Glycobiology.

[bib10] Cosson P. (2005). Dynamic transport of SNARE proteins in the Golgi apparatus. Proc. Natl. Acad. Sci. U. S. A..

[bib11] Hassinen A. (2010). Golgi <em>N</em>-Glycosyltransferases form both homo- and heterodimeric enzyme complexes in live cells *. J. Biol. Chem..

[bib12] Hossler P., Mulukutla B.C., Hu W.-S. (2007). Systems analysis of N-glycan processing in mammalian cells. PLoS One.

[bib13] Hutter S. (2017). Glycosylation flux analysis reveals dynamic changes of intracellular glycosylation flux distribution in Chinese hamster ovary fed-batch cultures. Metab. Eng..

[bib14] Jimenez del Val I., Nagy J.M., Kontoravdi C. (2011). A dynamic mathematical model for monoclonal antibody N-linked glycosylation and nucleotide sugar donor transport within a maturing Golgi apparatus. Biotechnol. Prog..

[bib15] Khoder-Agha F. (2019). Assembly of B4GALT1/ST6GAL1 heteromers in the Golgi membranes involves lateral interactions via highly charged surface domains. J. Biol. Chem..

[bib16] Krambeck F.J. (2009). A mathematical model to derive N-glycan structures and cellular enzyme activities from mass spectrometric data. Glycobiology.

[bib17] Krambeck F.J. (2017). Model-based analysis of N-glycosylation in Chinese hamster ovary cells. PLoS One.

[bib18] Kremkow B.G., Lee K.H., Glyco-Mapper (2018). A Chinese hamster ovary (CHO) genome-specific glycosylation prediction tool. Metab. Eng..

[bib19] Liu G., Neelamegham S. (2014). A computational framework for the automated construction of glycosylation reaction networks. PLoS One.

[bib20] McDonald A.G. (2014). Galactosyltransferase 4 is a major control point for glycan branching in N-linked glycosylation. J. Cell Sci..

[bib21] McDonald A.G. (2014). Galactosyltransferase 4 is a major control point for glycan branching in N-linked glycosylation. J. Cell Sci..

[bib22] McInnes L., Healy J. (2018).

[bib23] North S.J. (2010). Glycomics profiling of Chinese hamster ovary cell glycosylation mutants reveals N-glycans of a novel size and complexity. J. Biol. Chem..

[bib24] Rabouille C. (1995). Mapping the distribution of Golgi enzymes involved in the construction of complex oligosaccharides. J. Cell Sci..

[bib25] Reinhart D., Bioprocessing of Recombinant CHO-K1, CHO-DG44, CHO -S. (2019). CHO expression hosts favor either mAb production or biomass synthesis. Biotechnol. J..

[bib26] Schaub B.E. (2006). Transition of galactosyltransferase 1 from trans-Golgi cisterna to the trans-Golgi network is signal mediated. Mol. Biol. Cell.

[bib27] Shannon P. (2003). Cytoscape: a software environment for integrated models of biomolecular interaction networks. Genome Res..

[bib28] Spahn P.N. (2016). A Markov chain model for N-linked protein glycosylation – towards a low-parameter tool for model-driven glycoengineering. Metab. Eng..

[bib29] Tie H.C. (2018). The spatial separation of processing and transport functions to the interior and periphery of the Golgi stack. Elife.

[bib30] van der Maaten L., Hinton G. (2008). Viualizing data using t-SNE. J. Mach. Learn. Res..

[bib31] Veillon L. (2017). Characterization of isomeric glycan structures by LC-MS/MS. ELECTROPHORESIS.

[bib32] Velasco A. (1993). Cell type-dependent variations in the subcellular distribution of alpha-mannosidase I and II. JCB (J. Cell Biol.).

[bib33] Wainer J., Cawley G. (2021). Nested cross-validation when selecting classifiers is overzealous for most practical applications. Expert Syst. Appl..

[bib34] Walsh G. (2018). Biopharmaceutical benchmarks 2018. Nat. Biotechnol..

[bib35] Yang Z. (2015). Engineered CHO cells for production of diverse, homogeneous glycoproteins. Nat. Biotechnol..

[bib36] Yin B. (2015). Glycoengineering of Chinese hamster ovary cells for enhanced erythropoietin N-glycan branching and sialylation. Biotechnol. Bioeng..

