# Supplementary material for: CHOGlycoNET: Comprehensive glycosylation reaction network for CHO cells
Source: Metab Eng. 2023 Mar;76:87–96. doi: 10.1016/j.ymben.2022.12.009 (PMC11132536; doi:10.1016/j.ymben.2022.12.009)
Supplement: Multimedia component 1 [file mmc1.zip › SupplMaterial/SuplMat7_Clustering_Analysis.docx]

**Clustering of samples in the UMAP dimensionality**

Fig. 6 demonstrates a strong clustering of the samples based on their reaction network. Colouring the UMAP graph based on 1) the CHO cell lineage (CHO-K1 or CHO-S), 2) the lab from which the dataset originates or 3) the glycoprotein analysed in the samples. As shown in Fig. S1, none of the parameters seemed to dominate the clustering of the samples. A significant overlap between the samples of the different datasets-labs is observed, as shown in Fig. 1B. Interestingly, samples from the Borth lab can be found in all the different clusters, showcasing the diversity of the samples included in the Borth dataset. The different engineering modifications applied within each study, in addition to the fact that multiple proteins were considered in some of the studies (Fig. 1B) resulted to a highly diverse set of reaction networks, the UMAP representation of which was not found to be dependent on a single parameter.


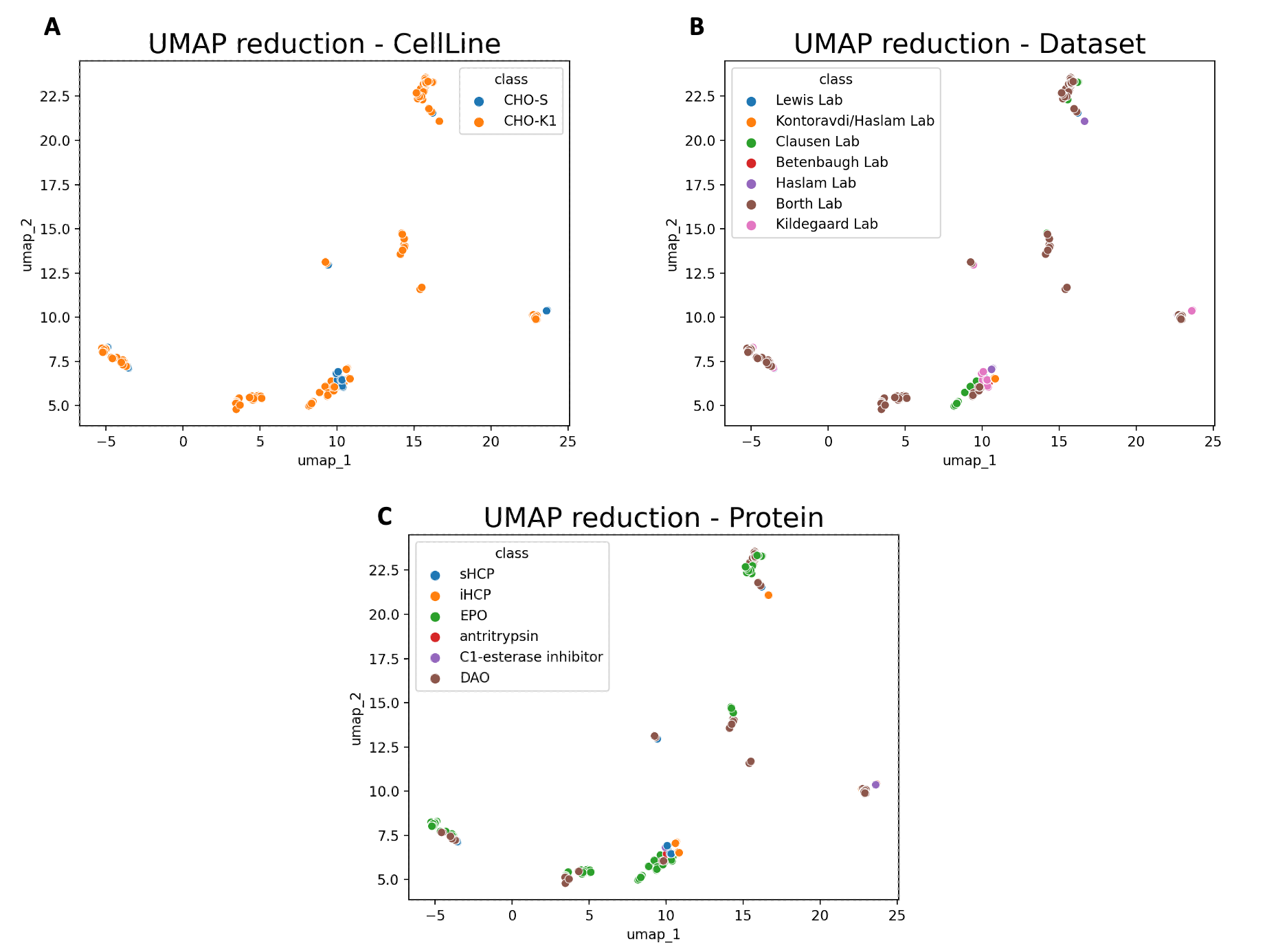


Figure S1. The UMAP representation of the samples coloured by A) the cell lineage, B) the dataset-lab from which the data originated and C) the protein analysed in the sample.
